# Feasibility of stereotactic radiotherapy for lung lesions and conventional radiotherapy for nodal areas in primary lung malignancies

**DOI:** 10.1186/s13014-018-1071-5

**Published:** 2018-07-11

**Authors:** Yeon Joo Kim, Su Ssan Kim, Si Yeol Song, Eun Kyung Choi

**Affiliations:** Department of Radiation Oncology, Asan Medical Center, University of Ulsan College of Medicine, 88 Olympic-ro 43-gil, Songpa-gu, Seoul, 05505 South Korea

**Keywords:** Lung cancer, Radiotherapy, Survival, Toxicity

## Abstract

**Background:**

Combined stereotactic body radiotherapy (SBRT) for lung lesions and conventional radiotherapy (CRT) for nodal areas may be more effective than CRT alone in patients with locally advanced lung cancer.

**Methods:**

This study included 21 patients with small primary lung tumors distant from the regional nodal areas. The SBRT dose was 40–60 Gy in 4 fractions. CRT doses were 66 Gy in 30 fractions for non-small cell lung cancer and 52.5 Gy in 25 fractions for small cell lung cancer.

**Results:**

The median follow-up duration was 12 months, and the median survival was 13 months. The 1 year overall survival, local recurrence-free survival, and distant metastasis-free survival rates were 60.5, 84.8, and 62.1%, respectively. Two patients experienced in-field local recurrence combined with out-field regional recurrence and/or distant failure. The major recurrence pattern was distant failure (crude incidence, 43%). Three patients aged ≥79 years experienced grade ≥ 3 acute radiation pneumonitis, and one also had idiopathic interstitial pneumonia.

**Conclusion:**

The combination of SBRT for the lung lesion and CRT for the nodal region seems to be effective and safe for lung malignancies. However, patients older in age and/or with underlying pulmonary disease require stricter lung dose constraints.

## Background

Definitive concurrent chemoradiotherapy (CCRT) has been recommended as the standard treatment for unresectable or medically inoperable stage II–III non-small cell lung cancer (NSCLC), based on the findings of randomized prospective trials [1–3]. Definitive CCRT is also the treatment of choice for limited-stage small cell lung cancer (LS-SCLC), as indicated by two previous meta-analyses [4, 5]. The common radiotherapy dose regimens include conventional radiotherapy (CRT) with 60–66 Gy (1.8–2 Gy/fraction once daily) for NSCLC and 45 Gy (1.5 Gy/fraction twice daily) or higher doses (60–70 Gy in 2 Gy/fraction once daily) for small cell lung cancer (SCLC).

**Fig. 1:**
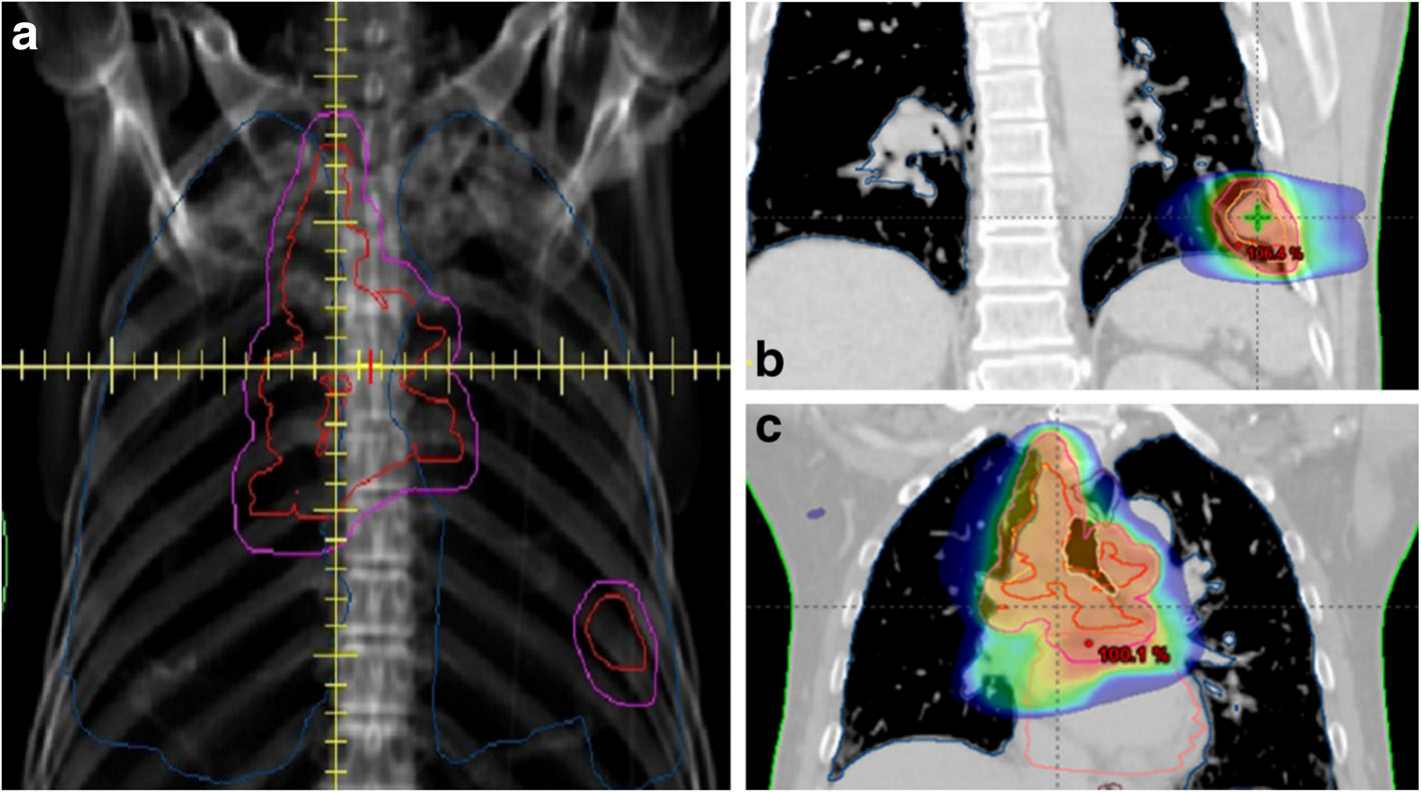
Anterior digitally reconstructed radiography (**a**) and dose distributions in a 65-year-old man treated with stereotactic body radiotherapy for a 2.4 cm-sized primary tumor in the left lower lobe (**b**), and with conventional radiotherapy for metastatic mediastinal lymph nodes (**c**)

As the primary lung lesion is located adjacent to the mediastinum and hilar lymph node (LN) region in most lung cancer patients, similar fractionation schemes are generally used for primary lung tumors and metastatic LNs. Some patients, however, are diagnosed with an isolated primary lesion, relatively small in size and distant from the nodal area (Fig. 1). Although relatively few patients present with these tumors, integrating the two separate target volumes is often difficult even when planning target volume (PTV) margins are larger. In contrast to the concept that all tumors should receive the same radiation regimens, different radiotherapy regimens could be administered to each target in these patients.

Stereotactic body radiotherapy (SBRT) consists of the administration of one or a few fractions of radiation (with high doses in each fraction), and is expected to achieve improved local control. Several reports indicate the effectiveness of SBRT in the management of patients with early-stage NSCLC, with 2–3 year local control rates of approximately 90% in patients with T1–2 disease [6–8]. In addition, two randomized trials compared SBRT with CRT in patients with medically inoperable stage I NSCLC. The SPACE trial reported that the two regimens resulted in similar progression-free survival and overall survival (OS) rates [9], whereas the CHISEL trial found that SBRT resulted in superior local control and OS [10]. The results of the CHISEL trial suggest that administration of SBRT to the lung lesion and CRT to the nodal area could improve control of the primary lung tumor and further improve control of the entire region in advanced lung cancer patients with two distinct target volumes.

This strategy might also reduce toxicities, owing to the reduction in PTV margins and the characteristics of SBRT including high targeting accuracy and rapid dose falloff. Because the SPACE trial found that the rates of pulmonary and esophageal toxicities were lower with SBRT than with CRT [9], our center treated these patients by administering SBRT to the lung nodule and CRT to the nodal area, a regimen termed stereotactic plus conventional radiotherapy (S + CRT). This retrospective analysis assessed the efficacy and safety of S + CRT.

## Methods

### Patients

The records of patients diagnosed with lung malignancy and treated with S + CRT at our hospital between March 2009 and July 2017 were retrospectively reviewed. All patients who received S + CRT had a small isolated primary lesion located distant from the nodal area (Fig. 1). Patients with distant metastases and those diagnosed with a primary cancer other than lung cancer within the previous 5 years were excluded. Patients underwent extensive examination, including pathologic confirmation, chest computed tomography (CT), 18-fluoro-deoxyglucose positron emission tomography (FDG-PET-CT), and/or brain magnetic resonance imaging. LNs of diameter ≥ 1 cm and definitive FDG uptake were classified as clinically malignant. LN metastases were pathologically confirmed whenever possible. Pulmonary function tests were performed prior to treatment. Data of all patients were reviewed, and tumor stage was determined according to the American Joint Committee on Cancer (AJCC) 7th edition TNM stage classification. SCLCs were also staged using the 7th AJCC system, based on the recommendations of the International Association for the Study of Lung Cancer [11]. The study protocol was approved by the institutional review board of our hospital, which waived the requirement for informed consent due to the retrospective nature of this study.

### Treatments

For SBRT planning, four-dimensional CT images (slice thickness, 2.5 mm) reflecting respiratory motion were acquired. The gross tumor volume (GTV) of the lung was delineated at the end-exhale phases using the lung setting (W = 1700, L = − 300). The internal target volume (ITV) was contoured using maximum intensity projection images, whereas the PTV was a 5 mm expansion of the ITV. The SBRT dose (range, 40–60 Gy) was administered in four fractions, as determined by the radiation oncologist. After SBRT, all patients underwent CT (slice thickness, 2.5 mm) for three-dimensional CRT planning. The GTV at the LN was contoured using the mediastinal setting (W = 600, L = 40), and the PTV margins were maintained as 7 mm radially and 10 mm longitudinally. The clinical target volumes were also delineated for some patients to cover the regional nodal areas in the same axial sections; however, smaller PTV margins (5 mm radially and 7 mm longitudinally) were considered in these patients. In patients treated with induction chemotherapy, the post-chemotherapy volume was defined as the GTV. Standard radiotherapy consisted of 66 Gy (2.2 Gy/fraction) for NSCLC and 52.5 Gy (2.1 Gy/fraction) for SCLC.

The organs at risk (OAR) included healthy lung tissues, the esophagus, the spinal cord, and the heart. The normal organ constraints included a maximal dose for the spinal cord of < 50 Gy, a mean lung dose (MLD) of < 20 Gy, a volume of the lung receiving at least 20 Gy (V20) of < 30%, a mean esophagus dose of < 35 Gy, and heart doses of V60 < 1/3, V45 < 2/3, and V40 < 100%.

Chest X-rays (CXR) were obtained weekly to monitor changes in tumor volume and any acute toxicities. Treatment was verified by weekly kV imaging guidance, using set-up correction based on carina and bony anatomy.

The main concurrent chemotherapy regimen for NSCLC consisted of weekly doses of paclitaxel combined with cisplatin or carboplatin. Patients with very large tumors and a high risk of radiation-related toxicities were initially treated with induction chemotherapy for volume reduction, with a regimen consisting of two cycles of gemcitabine plus cisplatin in sequential schedules every 3 weeks. Chemotherapy for SCLC consisted of intravenous etoposide and cisplatin administered every 3 weeks for four cycles, with radiotherapy initiated along with the third cycle of chemotherapy. Patients with a poor performance status or poor lung function were recommended to undergo radiotherapy alone.

### Follow-up, toxicity scoring, and statistical analysis

All patients underwent weekly complete blood counts and CXR. Patients were routinely followed up by a radiation oncologist and/or medical oncologist, with chest CT and/or CXR performed 1 month after treatment, every 3 months during the first 2 years, and every 6 months thereafter until 5 years after treatment.

The primary outcome was 1 year OS rate. Secondary endpoints included 1 year local recurrence-free survival (LRFS) and distant metastasis-free survival (DMFS) rates and toxicities. Toxicities were evaluated using the Common Terminology Criteria for Adverse Events version 4.03. Adverse events that occurred during treatment and within 3 months after CRT were defined as acute toxicities.

Follow-up durations were calculated from the date that treatment was discontinued. OS was calculated using the Kaplan-Meier method from the date of treatment discontinuation until death. Clinical and dosimetric factors of patients with and without grade ≥ 3 acute radiation pneumonitis (RP) were compared using Mann-Whitney U-tests. All statistical analyses were performed using SPSS version 21.0.

## Results

### Patients and treatments

A total of 21 patients were included; their demographic and clinical characteristics are shown in Table 1. Median patient age was 68 years (range, 52–88 years), and their initial or recurrent clinical stages were classified as stages IIA (*n* = 4), IIIA (*n* = 8), and IIIB (*n* = 9). All patients had forced expiratory volume in 1 s (FEV1) ≥40% of normal, making them eligible for the NPC 95–01 study [2]. Carbon monoxide diffusing capacity (DLco) was ≥60% in 17 patients, ≥40 and < 60% in three, and 37% in one. Eighteen patients had NSCLC, and three had SCLC, with 15 receiving definitive and six receiving salvage radiotherapy.

**Table 1:** Patient and treatment characteristics

Factors			Number (*n* = 21)
Sex		Male	17
		Female	4
Age (years)		Median (range)	68 (52–88)
Location (lobe)^a^		Right upper	5
		Right middle	3
		Right lower	4
		Left upper	6
		Left lower	7
T stage		1	13
		2	5
		3	3
N stage		1	4
		2	8
		3	9
Clinical stage		IIA	4
		IIIA	8
		IIIB	9
ECOG performance status		0	2
		1	16
		2	3
Weight loss		No	18
		Yes	3
Smoking history		No	4
		Yes	17
FEV1 (%)		Median (range)	83 (51–116)
DLco (%)^b^		Median (range)	73 (37–99)
Pathology		Squamous	8
		Adenocarcinoma	9
		Unknown NSCLC	1
		SCLC	3
Aim		Definitive	15
		Salvage	6
Radiotherapy dose (Gy)	SBRT	Median (range)	54 (40–60)
	CRT	Median (range)	60 (46–66)
Dose per fraction (Gy)	SBRT	Median (range)	14 (10–15)
	CRT	Median (range)	2 (2–6)
GTV (cc)	SBRT	Median (range)	6.4 (1.2–29.2)
	CRT	Median (range)	14.4 (0.9–104.8)
PTV (cc)	SBRT	Median (range)	27.7 (8.2–89.6)
	CRT	Median (range)	144.8 (27.7–404.1)
Chemotherapy		None	9
		Neoadjuvant	5
		Concurrent	4
		Both	3

*Abbreviations*: *ECOG* Eastern Cooperative Oncology Group, *FVC* forced vital capacity, *FEV1* forced expiratory volume in 1 s, *DLco* carbon monoxide diffusing capacity, *SBRT* stereotactic body radiotherapy, *CRT* conventional radiotherapy, *NSCLC* non-small cell lung cancer, *SCLC* small cell lung cancer, *GTV* gross tumor volume, *PTV* planning target volume

^a^Two patients had two lung nodules each, and one presented with three lung nodules

^b^DLco information not available for four patients

The median SBRT dose was 54 Gy (range, 40–60 Gy), which was administered in 4 fractions (median fraction size, 14 Gy; range, 10–15 Gy/fraction). Eight patients who received 60 Gy over 4 fractions underwent treatment twice a week, whereas the others were treated daily. Seven patients were treated with SBRT using three-dimensional conformal RT (3D-CRT), whereas 13 received volumetric arc therapy. Non-isocentric technique using a Cyberknife was applied to one patient. Median time interval between the start of SBRT and the start of CRT was 8 days (range, 6–24 days). The median CRT dose was 60 Gy (range, 46–66 Gy), and the median dose per fraction was 2 Gy (range, 2–6 Gy). Only one patient received 6 Gy fractions, which is strictly defined as hypofractionated radiotherapy, rather than CRT. Nevertheless, because this study focused on the difference in fractionation regimens administered to the lung and nodal area, this patient was included. Four patients received intensity modulated RT, and the others received 3D-CRT. Nine patients did not receive any chemotherapy due to old age (≥75 years) and comorbidities (*n* = 6), including idiopathic interstitial pneumonia (IIP) (*n* = 1), chronic obstructive pulmonary disease (COPD) (n = 1), and patient refusal (n = 1).

### Survival and patterns of failure

**Fig. 2:**
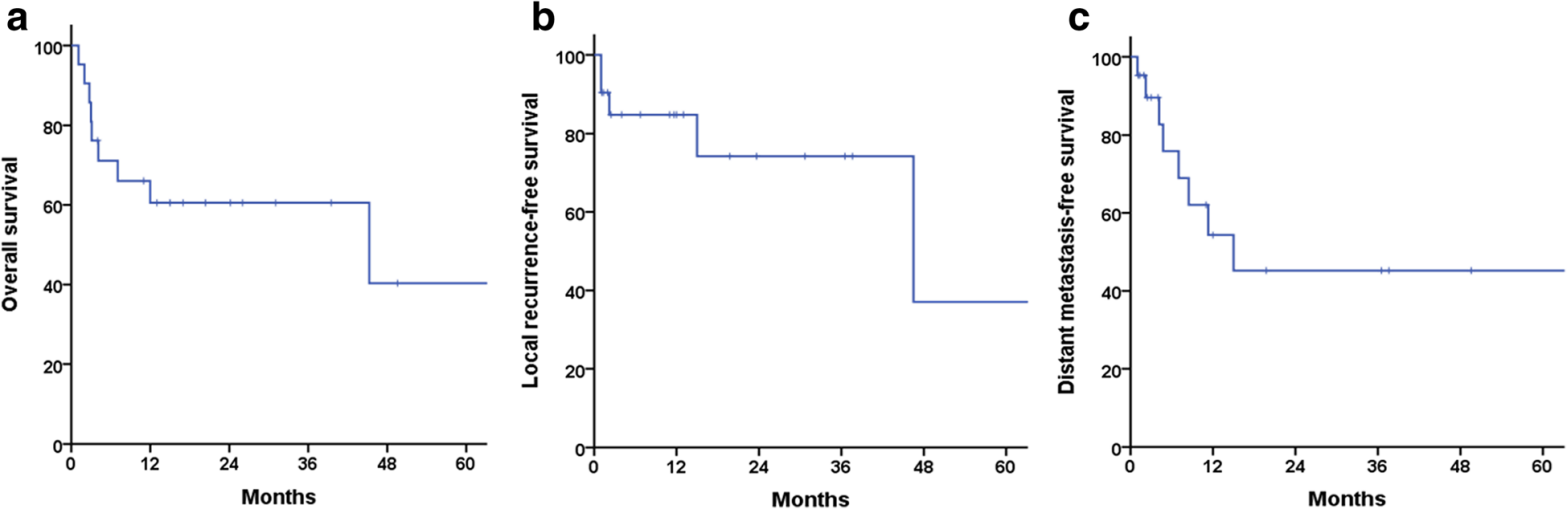
Kaplan-Meier graphs showing (**a**) overall survival, (**b**) local recurrence-free survival, and (**c**) distant metastasis-free survival in the study cohort

The median follow-up duration was 12 months (range, 1–92 months). Median survival was 13 months (range, 1–92 months). The 1 year OS rate was 60.5% (Fig. 2a), whereas the 1 year LRFS and DMFS rates were 84.8 and 62.1%, respectively (Fig. 2b and c). The 2 year OS rate was also 60.5%, whereas the 2 year LRFS and DMFS rates were 74.2 and 45.2%, respectively.

Two patients experienced in-field local recurrence, combined with out-field regional and/or distant failure. The locations of the in-field recurrences were the mediastinal and low cervical nodal areas. Both patients had NSCLC, with one having recurrence after lobectomy. Neither received any chemotherapy. Five patients exhibited out-field regional failure (crude incidence rate, 24%), with three simultaneously diagnosed with distant recurrence. All out-field regional recurrences occurred in nodal areas, not in lung fields, and all of these patients had NSCLC. Four of these patients received definitive treatment, whereas one received salvage treatment. Three did not receive any chemotherapy. The major recurrence pattern was distant failure, which occurred in nine patients (crude incidence, 43%). Of these nine patients, only one had SCLC, two received salvage treatment, and two did not receive chemotherapy.

### Toxicity

Nine patients experienced acute radiation esophagitis, including eight with grade 2 disease and one with grade 3 disease. Except for one patient who finally progressed to grade 3 late radiation esophagitis, the other patients were successfully managed medically. Eight patients experienced acute RP, including five with grade 2, one with grade 3, and two with grade 4 (life-threatening) disease. The characteristics of the three patients with acute RP of grade ≥ 3 are summarized in Table 2. All these patients were aged ≥79 years.

**Table 2:** Characteristics of three patients with grade ≥ 3 acute radiation pneumonitis

Factors		Patients		
Radiation pneumonitis grade		3	4	4
Sex		Male	Male	Male
Age (year)		88	79	80
ECOG performance status		2	1	1
Pre-treatment pulmonary function test	FVC (L)	2.5	2.1	4.8
	FEV1 (L)	1.9	2.0	2.9
	DLco (%)	85	37	84
Combined pulmonary disease		None	IIP	None
Aim		Definitive	Salvage	Salvage
SBRT dose (Gy/fraction)		48/4	40/4	60/4
CRT dose (Gy/fraction)		66/33	60/10	60/30
GTV (cc)	Lung	29.2	6.1	6.4
	Nodal area	17.7	4.9	24.2
PTV (cc)	Lung	89.6	26.5	30.1
	Nodal area	298.2	27.7	361.0
Chemotherapy		None	None	None
Occurrence time of radiation pneumonitis (days)		40	43	18
Bilateral or unilateral lung involvement of radiation pneumonitis		Unilateral	Bilateral	Bilateral
Time from onset of symptoms to death (days)		53	17	15

*Abbreviations*: *ECOG* Eastern Cooperative Oncology Group, *FVC* forced vital capacity, *FEV1* forced expiratory volume in 1 s, *DLco* carbon monoxide diffusing capacity, *SBRT* stereotactic body radiotherapy, *CRT* conventional radiotherapy, *GTV* gross tumor volume, *PTV* planning target volume, *IIP* idiopathic interstitial pneumonia

One patient who initially had IIP showed poor lung function with a DLco of 37%; this patient was treated with hypofractionated radiotherapy to the nodal area (60 Gy over 10 fractions). Nevertheless, as the PTV of the nodal area was very small (27.7 cc), more conservative dose constraints were applied. Dosimetric parameters showed that his MLD was 7.1 Gy and his V20 was 9.1% (Table 3). All other patients satisfied the institutional OAR dose constraints.

**Table 3:** Dosimetric parameters for lungs in all patients

Patients	MLD (Gy)	V5 (%)	V20 (%)	MILD (Gy)
1	12.8	53.0	22.7	18.7
2	4.0	20.6	4.6	5.4
3	15.9	72.2	27.2	20.8
4^a^	17.0	55.7	28.0	28.5
5^a^	7.1	42.8	9.1	7.2
6	18.3	80.3	32.9	14.1
7	18.8	74.0	37.4	27.0
8^a^	16.4	78.3	29.6	15.2
9	14.9	47.5	25.9	20.6
10	10.3	25.5	18.8	18.9
11	13.5	48.5	22.7	17.1
12	13.3	54.1	25.9	21.0
13	12.1	60.9	19.0	17.3
14	11.1	49.1	19.8	20.8
15	13.2	48.1	20.3	22.1
16	7.9	29.8	13.8	15.9
17	11.6	50.4	18.9	17.3
18	13.5	47.7	27.4	26.5
19	14.7	64.3	23.1	22.0
20	3.4	17.0	3.2	5.3
21	4.9	26.0	5.3	8.0

*Abbreviations*: *MLD* mean lung dose, *VX* volume of lung receiving at least X Gy, *MILD* mean ipsilateral lung dose

^a^Patients with grade ≥ 3 acute radiation pneumonitis

The only significant factor associated with grade ≥ 3 RP was older age (*p* = 0.011). Other demographic and clinical factors, including sex, performance status, smoking history, pre-treatment pulmonary function test results, aim of radiotherapy (definitive or salvage), and combined chemotherapy, were not significantly associated with grade ≥ 3 RP. Due to the small number of patients enrolled, we could not reliably determine the dosimetric parameters, such as MLD, V5, V20, mean ipsilateral lung dose, and PTV size, predictive of grade ≥ 3 RP. Two patients without a history of acute RP experienced late RP of grade 2. None of the patients experienced any grade ≥ 2 hematologic toxicity.

## Discussion

The present study describes the clinical outcomes in patients who underwent S + CRT. Definitive CCRT for unresectable or medically inoperable stage II–III NSCLC has shown local failure rates of 30–53% [1, 2] and a 3 year OS rate of approximately 27% [12]. Moreover, CCRT for LS-SCLC has shown a 2 year intrathoracic recurrence rate of 66% [5] and a 3 year OS rate of 14% [4]. The present study found that the 2 year LRFS rate was 74.2%, the crude local failure rate was 24%, and the 2 year OS rate was 45.2%, consistent with the findings of previous studies.

Only two of our patients experienced in-field recurrences at nodal areas alone. A previous study investigated the patterns of loco-regional failure in locally advanced NSCLC patients who received definitive CCRT (66 Gy in 24 fractions) [13]. There were about a 2.5-fold higher absolute risk of primary tumor failure (16%) than nodal failure (6%). In the present study, we observed no recurrence in primary lung lesions which may be attributed to the administration of SBRT. The results of a randomized trial comparing SBRT and CRT in patients with inoperable stage I NSCLC (CHISEL) also support our findings, with both longer LRFS (hazard ratio [HR] = 0.29, *p* = 0.002) and OS (HR = 0.51, *p* = 0.02) in the SBRT arm [10]. Although it may be worrisome that reducing PTV margins resulted in recurrences in the non-irradiated lung field between the PTVs of the primary lung tumor and LNs, there were no recurrences in any lung tissue. Because most regional recurrences occurred in nodal areas, it is important to improve nodal disease control. Although doses have been escalated to achieve greater local control, the RTOG 0617 trial, which compared regimens of 74 and 60 Gy (2 Gy/fraction), found that the higher dose regimen did not increase 2 year LRFS and OS rates [14]. This finding suggested that dose escalation in CRT may not be sufficient for improving tumor control, suggesting that SBRT may provide additional benefits.

Attempts have been made to administer SBRT to patients with locally advanced cancers. For example, a prospective, single-institution study evaluated the feasibility of CRT (59.4 Gy in 33 fractions) followed by SBRT (20 Gy in 2 fractions or 19.5 Gy in 3 fractions) in patients with stage II–III NSCLC and residual disease on positron emission tomography [15]. Over a median follow-up of 13 months, the local control rate was found to be 83%, similar to our crude local control rate of 76%. Additional studies are required to determine whether administration of SBRT to both lung lesions and LNs reduces the incidence of nodal in-field recurrences.

Patients administered S + CRT are likely to experience the abscopal effect of SBRT [16]. Because SBRT in patients with stage I NSCLC was performed in the absence of nodal staging and irradiation, it should result in higher rates of regional recurrence than with surgery. However, regional failure rates in patients undergoing SBRT were comparable to those in patients undergoing surgery, including LN dissection [6, 8]. This phenomenon may be due to an abscopal effect, defined as the anti-tumor effect of radiation outside the radiotherapy field, resulting from increased tumor antigen expression and T-cell response following radiotherapy [16]. Although additional evidence is needed, the combination of CRT and SBRT may improve oncologic outcomes in patients with lung cancer through abscopal effect.

Previous studies of patients with NSCLC treated with 63–66 Gy in fractions of 1.8–2 Gy reported grade ≥ 3 pulmonary toxicities presenting as acute complications in 4–5% of patients and late toxicities in 11% [1, 2], and a prospective, single-institution study reported an incidence rate of grade ≥ 3 acute RP of 11% [15]. Acceptable rates of severe toxicity of SBRT in patients with stage I NSCLC have been reported to range from 4.5 to 10.2% [17]. We suggest that toxicity rates may be lower with SBRT than with CRT, as SBRT has high targeting accuracy and rapid dose falloff. Moreover, the greater accuracy of SBRT resulted in smaller PTV margins. A randomized trial comparing SBRT and CRT in patients with inoperable stage I NSCLC (SPACE trial) reported a significantly lower rate of esophagitis (8% vs. 30%, *p* = 0.006) and a lower rate of RP (19% vs. 34%, *p* = 0.26) in the SBRT than in the CRT group [9]. Although the incidence rate of grade ≥ 3 acute RP in the present study was 21%, higher than in previous trials, our study population was older, with a median age of 68 years (compared with median ages of 61–63 years in previous studies), and included patients with recurrent disease after curative surgery. As age ≥ 60 years is a predictive factor for RP [18], our patients had a greater likelihood of developing RP.

One patient in the present study with grade 4 RP had IIP and a DLco of only 37%. A more pronounced decrease in lung function has been reported in individuals with an initial DLco of ≤50% [19]. Lung constraints in the present study included MLD < 20 Gy and V20 < 30%. A study assessing the association of dose-volume parameters with the risk of grade ≥ 3 RP found that patients who satisfied the threshold dose-volume histogram curve, defined by V20 ≤ 25%, V25 ≤ 20%, V35 ≤ 15%, and V50 ≤ 10%, had an incidence of grade ≥ 3 RP of only 2% [20]. Stricter lung constraints are therefore needed for older aged patients and those with poor lung function.

This study had several limitations, including its retrospective design, the small number of patients, and the heterogeneity of the enrolled patients. Although CCRT was the standard treatment, 43% of these patients did not receive any chemotherapy. Therefore, the results of the present study are not representative of standard populations. The potential numbers of candidates for S + CRT are limited, because relatively few patients present with primary lung lesions distinct from nodal areas.

## Conclusions

This retrospective analysis of lung cancer patients treated with S + CRT showed promising local control and survival rates, as well as acceptable toxicities. Stricter lung dose constraints are need for older aged patients and those with underlying pulmonary diseases. Additional studies, in larger numbers of patients and with stricter enrollment criteria, are required to confirm these findings.

## Abbreviations

3D-CRT: Three-dimensional conformal RT
AJCC: American Joint Committee on Cancer
CCRT: Concurrent chemoradiotherapy
COPD: Chronic obstructive pulmonary disease
CRT: Conventional radiotherapy
CT: Computed tomography
CXR: Chest X-rays
DLco: Carbon monoxide diffusing capacity
DMFS: Distant metastasis-free survival
FDG-PET-CT: 18-fluoro-deoxyglucose positron emission tomography
FEV1: Forced expiratory volume in 1 s
GTV: Gross tumor volume
HR: Hazard ratio
IIP: Idiopathic interstitial pneumonia
ITV: Internal target volume
LN: Lymph node
LRFS: Local recurrence-free survival
LS-SCLC: Limited-stage small cell lung cancer
MILD: Mean ipsilateral lung dose
MLD: Mean lung dose
NSCLC: Non-small cell lung cancer
OAR: Organs at risk
OS: Overall survival
PTV: Planning target volume
RP: Radiation pneumonitis
S + CRT: Stereotactic plus conventional radiotherapy
SBRT: Stereotactic body radiotherapy
SCLC: Small cell lung cancer
V20: Volume of lung receiving at least 20 Gy
